# The impact of drought on wheat leaf cuticle properties

**DOI:** 10.1186/s12870-017-1033-3

**Published:** 2017-05-08

**Authors:** Huihui Bi, Nataliya Kovalchuk, Peter Langridge, Penny J. Tricker, Sergiy Lopato, Nikolai Borisjuk

**Affiliations:** 1 Australian Centre for Plant Functional Genomics, PMB1 Glen Osmond, Adelaide, South Australia 5064 Australia; 2 School of Agriculture, Food and Wine, University of Adelaide, PMB1 Glen Osmond, Adelaide, South Australia 5064 Australia; 3 Present address: School of Life Sciences, Huaiyin Normal University, Huaian, 223300 China

**Keywords:** Cuticular wax, β-diketone, Glaucousness, Residual transpiration rate, Stomatal density, *Triticum aestivum*

## Abstract

**Background:**

The plant cuticle is the outermost layer covering aerial tissues and is composed of cutin and waxes. The cuticle plays an important role in protection from environmental stresses and glaucousness, the bluish-white colouration of plant surfaces associated with cuticular waxes, has been suggested as a contributing factor in crop drought tolerance. However, the cuticle structure and composition is complex and it is not clear which aspects are important in determining a role in drought tolerance. Therefore, we analysed residual transpiration rates, cuticle structure and epicuticular wax composition under well-watered conditions and drought in five Australian bread wheat genotypes, Kukri, Excalibur, Drysdale, RAC875 and Gladius, with contrasting glaucousness and drought tolerance.

**Results:**

Significant differences were detected in residual transpiration rates between non-glaucous and drought-sensitive Kukri and four glaucous and drought-tolerant lines. No simple correlation was found between residual transpiration rates and the level of glaucousness among glaucous lines. Modest differences in the thickness of cuticle existed between the examined genotypes, while drought significantly increased thickness in Drysdale and RAC875. Wax composition analyses showed various amounts of C31 β-diketone among genotypes and increases in the content of alkanes under drought in all examined wheat lines.

**Conclusions:**

The results provide new insights into the relationship between drought stress and the properties and structure of the wheat leaf cuticle. In particular, the data highlight the importance of the cuticle’s biochemical makeup, rather than a simple correlation with glaucousness or stomatal density, for water loss under limited water conditions.

**Electronic supplementary material:**

The online version of this article (doi:10.1186/s12870-017-1033-3) contains supplementary material, which is available to authorized users.

## Background

Bread wheat, *Triticum aestivum*, is the world’s most widely grown crop, representing about 30% of the cereal cultivation area and providing 20% of the calories for the human population. Drought significantly limits crop production and the duration of drought periods is increasing due to climate change, declining ground and surface water resources, and warming of air temperature in most of the cereal cropping regions around the world [1]. An understanding of the physiological, biochemical, and genetic mechanisms allowing plants to cope with environmental challenges is of vital importance for breeding crops with improved stress tolerance and performance under stress [2].

The plant cuticle evolved as an exterior extension of epidermal cell walls, is a continuous hydrophobic sheet that covers the aerial surfaces of all plant organs and acts as an interface in plants’ interactions with various biotic and abiotic factors. The cuticle is a crucial barrier that, in concert with stomata, controls plant water status and helps plants survive under drought and high UV radiation [3]. Structurally, the wheat cuticle is a 0.1–10 μm thick membrane composed principally of a polyester matrix intertwined with a range of long chain hydrocarbons. Based on solubility in organic solvents, the cuticle components are divided into insoluble cutin and soluble cuticular waxes. Intracuticular wax is embedded in the underlying cutin polyester framework and epicuticular wax is overlaid on the cutin matrix and intracuticular wax. The waxes are typically a complex mixture of derivatives of very-long-chain saturated aliphatics, which include fatty acids, alkanes, aldehydes, ketones, and primary and secondary alcohols [4, 5].

Many elements of the cuticle biosynthesis pathways, including cuticle-related genes encoding key enzymes and regulatory transcription factors, were uncovered primarily by characterising cuticle mutants in *Arabidopsis*, tomato, rice, maize, and barley [6–8]. The biosynthesis of major cuticle constituents begins with de novo synthesis of C16-C18 fatty acids in the plastids of epidermal cells. The fatty acids are converted to acyl-CoAs prior to their export to the endoplasmic reticulum, where they are transformed into very long chain fatty acyl-CoAs (VLCFAs) by the fatty acid elongation complex (FAE). These saturated VLCFAs containing 22–34 carbons can be released from the FAE as free fatty acids or serve as precursors for (i) the alcohol-forming pathway for conversion into even-numbered primary alcohols and alkyl esters, for (ii) the alkane-forming pathway which yields aldehydes, odd-numbered alkanes, secondary alcohols and ketones, and for (iii) the β-diketone pathway which is less prevalent than the other two pathways but represents an important pathway in some plants like wheat and barley [9, 10].

It has been proposed that the main physiological role of the cuticle is the reduction of water loss. The cuticle delays the onset of cellular dehydration stress under drought and is therefore considered an important component of protection from drought [5, 11, 12]. During water deficit, stomata close and nanoscale diffusion pathways crossing the cuticle become the primary path of plant water loss. Non-stomatal water loss through the leaf epidermis may account for up to 50% of total loss in drought-stressed wheat plants during the day and 60% during the night [13].

Accumulation of epicuticular waxes on plant surfaces often results in a bluish-white colouration termed glaucousness, which is a visible form of densely distributed epicuticular wax crystalloids. Glaucousness is formed in wheat due to the presence of C31 β-diketones on the surfaces of aerial organs [14–17]. Recently, the genes encoding three key enzymes involved in the synthesis of β-diketones have been reported in wheat and barley [10, 18]. Glaucousness increases radiation reflectance and reduces leaf temperatures and transpiration, thereby enhancing leaf survival under water stress [19, 20]. As a classical genetic marker and agronomic trait, glaucousness has been intensively studied in association with drought/heat tolerance and yield in different wheat varieties [21–24]. However, the precise value of this trait in relation to maintenance of plant biomass and grain yield under stress remains uncertain because of the complex biochemical and genetic nature of this easily visible trait.

It has been assumed that the amount and specific biochemical makeup of the waxes to a large extent define protective functions of the cuticle [25, 26]. Increased amounts of cuticular waxes in transgenic plants have been associated with improved drought tolerance in plants such as *Arabidopsis* [27], alfalfa [28] and Camelina [29]. Breeding barley for higher tolerance and yield under drought led to increased amounts of cuticular waxes, further confirming a connection between drought tolerance and the cuticle [30]. Another example is the *glossy 1–2* (*gl1–2*) mutant in rice with reduced accumulation of alkanes, aldehydes and fatty acids. The *gl1–2* mutant plants exhibited increased drought sensitivity and cuticular permeability at the reproductive stage [31].

The genetic, physiological and metabolic determinants influencing crop yield and drought tolerance have been studied in five Australian wheat lines, Kukri, Excalibur, Drysdale, RAC875 and Gladius [22, 32–36]. Kukri has a lower level of drought tolerance than the other four lines which represent important sources of drought tolerance in the southern Australian environment. Kukri was used as the drought-sensitive parent in several mapping populations for drought and yield related studies [33]. Most recently, two of these lines, Kukri and RAC875, have been investigated for cuticle composition and its regulation by drought-responsive transcription factors of the MYB family [16].

In this work, the five wheat lines were used to: (i) examine leaf cuticle properties as a water loss barrier for wheat plants grown under two different watering regimes, (ii) uncover the stomatal and cuticle-related characteristics responsible for differences in water loss and the responses of these traits to drought, and (iii) establish possible links between cuticle composition, permeability and drought tolerance of these wheat lines.

## Methods

### Plant, growth and sampling

The five genotypes used in this study were all bred for the southern Australian environment where yields are generally in the range of 1 to 4 t/ha. Under favourable conditions, where average yields are over 2 t/ha, all show a similar yield but in more severe environments, Kukri is the poorest performer [33, 37]. There are also differences between genotypes in their general response to drought stress. Drysdale was selected based on water use efficiency measured through carbon isotope discrimination but responds poorly to the saline and low nutrient soils in much of southern Australia. RAC875 and Excalibur differ in their physiological response to drought stress with RAC875 showing a constitutive response while Excalibur displays an adaptive mechanism [22]. Gladius is the most recent of the varieties, released in 2007, and has both RAC875 and Excalibur in its parentage (http://pbr.ipaustralia.plantbreeders.gov.au/).Therefore, these genotypes provide a spectrum of lines that have been selected for performance in a similar environment but differ in their stress responses. Drysdale, Excalibur, Gladius and Kukri are released varieties and RAC875 is a breeding line available, with permission, from the University of Adelaide and Australian Grain Technologies. Drysdale was developed by the CSIRO in conjunction with NSW Agriculture. Excalibur and Kukri were developed by the University of Adelaide Roseworthy Campus Wheat Breeding Team, and Gladius was developed by Australian Grain Technologies’ Roseworthy Campus Wheat Breeding Program. All seeds used in these experiments were from our own collection. Drysdale was originally sourced for our collection from AWB seeds Ltd.

Sixteen seeds (8 seeds/subplot) of each of the five selected genotypes: Kukri, Excalibur, Drysdale, RAC875 and Gladius, were sown in each of two large containers (112 × 76 × 50 cm) in a glasshouse, with a 14 h day/10 h night cycle. The two containers shared the same distribution of seeds and a randomised block design with 10 subplots distributed within each container exactly as described by Amalraj et al. [38]. Each container accommodated 80 tested plants flanked by a border row (var. Gladius) on each short side of the bin. One container was used for each of the controlled well-watered and drought conditions with drought imposed as shown in Additional file 1. To maintain and monitor water status, containers were equipped with an automatic watering system and four soil water tensiometers (gypsum blocks) installed at 10 cm and 30 cm soil depths, and connected to a data logger for continuous monitoring of the soil water tension. Watering in the drought bin was terminated 32 days after planting when plants were at stem elongation stage, while continuous watering was applied to the well-watered bin. The soil water tension in the drought bin gradually and slowly increased and flag leaves for all the experiments conducted in this study were sampled after the reading of 30 cm tensiometer reached the highest measureable level of 600 kPa in the drought treatment (for details see Additional file 1). Air-conditioning in the glasshouse controlled temperature fluctuations and the temperatures, recorded every 15 min, were 19.9 ± 3.3 °C (mean ± standard deviation) for the period from planting to sampling of flag leaves for all the analyses. Flag leaves were selected for all the analyses, as they are the main source of assimilates for grain development.

### Cuticle permeability assay

Flag leaves were detached from plants grown in the well-watered container or drought container 20 days after anthesis, and leaf weight was immediately measured using an analytical balance (OHAUS Corporation, Parsippany, USA) before being placed into a collection tube. Leaves in the opened collection tubes were dehydrated at room temperature (23 °C) for 12 h with leaf weight measured every hour. Leaves were then dried in a 37 °C incubator for 72 h after which leaf weight was measured and taken as the dry weight.

### Leaf surface imprints

The leaf surface imprint method, modified from Sinclair and Dhingra [39], was used to estimate stomatal numbers per unit area. Flag leaves from the main tiller were detached 20 days after anthesis from five plants of each cultivar grown under well-watered conditions or drought, and the mid-point between the major vein and the leaf margin and half-way along the long axis of the leaf were used to make impressions of the surfaces. Cyanoacrylate adhesive (Supa glue; Selleys, Padstow, NSW, Australia) was applied to the area and the glued side was placed against a microscope slide. A second slide was placed on top of the leaf in the same direction. The slides were held together with two large bulldog clips for 3 minutes, after which the two slides were separated and the leaf was peeled off. Leaf imprints were examined under the Leica AS LMD microscope (Leica Microsystems, Wetzlar, Germany) with differential interference contrast (DIC) at 200 times magnification at the Waite Facility of Adelaide Microscopy. Stomatal counts were made from each of three images of each of the imprints from five biological replicates.

### Transmission electron microscopy (TEM)

Flag leaf blades were collected at day 24 after anthesis and several segments close to the major vein of approximately 5 mm × 3 mm in size were cut from the mid-point of a leaf from each of three plants. In a fume hood, cut pieces were placed into TEM fixative [4% sucrose, 1× PBS (phosphate buffered saline), 4% paraformaldehyde, and 0.25% glutaraldehyde]. After overnight incubation at 4 °C, samples were rinsed twice with 1 × PBS at 4 °C to remove fixative. Then samples were dehydrated in an ethanol series: 50%, 70%, 90%, 95% and 100%, each change after approximately 1 hour, followed by two changes of 100% dry ethanol (on a molecular sieve). Samples were stored in 100% dry ethanol at 4 °C until resin infiltration. Samples were first infiltrated in a 50:50 mix of 100% ethanol and LR White Resin (London Resin Co. Ltd., London, UK) for 8 hours, followed by three changes of 100% LR White Resin, also for 8 hours each. Next, infiltrated specimens were embedded in fresh 100% LR White Resin in gelatin capsules. Finally, embedded samples were polymerised at 60 °C for 24 h. Prior to sectioning on the ultramicrotome, resin blocks were trimmed with a razor blade to make a trapezoid face. 70 nm thick sections were generated using a diamond knife. Sections were first stained with 4% uranyl acetate for 20 min, then with lead citrate for 20 min after three rinses in water. After another three rinses in water and air drying, sections were examined under a Philips CM100 transmission electron microscope at the Adelaide Microscopy Waite Facility. Samples from one or two plants of each genotype were subjected to TEM.

### Scanning electron microscopy (SEM)

The epicuticular wax was examined using a scanning electron microscope in the Adelaide Microscopy Unit (https://www.adelaide.edu.au/microscopy/, University of Adelaide, Australia). Several segments of approximately 4 mm × 3 mm were cut from a similar location on the same leaf as the TEM samples and examined as described by Bi et al. [16] under a Philips XL30 Field Emission Scanning Electron Microscope, equipped with a Gatan CT1500 HF Cryo-transfer Stage.

### Composition analysis of cuticular waxes

For the wax composition analysis, the 6.5 cm long basal portions of the flag leaf blades from each of three individual plants were collected 24 days after anthesis. The weight of each leaf section was measured, and leaves were snap frozen in liquid nitrogen and further stored at −80 °C until wax extraction. Wax extraction and GC-MS analysis were carried out following the same procedures as described by Bi et al. [16]. GC-MS analysis was conducted in the W.M. Keck Metabolomics Research Laboratory of Iowa State University (USA) according to the procedure described by Cha et al. [40].

### Statistical analysis of data

Data on stomatal density and wax composition were analysed using two-way ANOVA with the Fisher’s least significant difference post-hoc test in GenStat (16th Edition; VSN International Ltd., Hemel Hempstead, UK). The statistical significance of differences in water loss rates was analysed using a General Linear Model repeated measures ANOVA in IBM^©^ SPSS^©^ Statistics v. 24 within and between subjects with post hoc multiple comparisons for the least significant difference between genotypes at α0.05. Carbon chain length distributions of major cuticular waxes were analysed using IBM^©^ SPSS^©^ Statistics v. 24 General Liner Model multivariate analyses with treatment and genotype fixed factors in the model. Post hoc multiple comparisons between genotypes were performed as before.

## Results

### Examination of leaf surface permeability

**Fig. 1:**
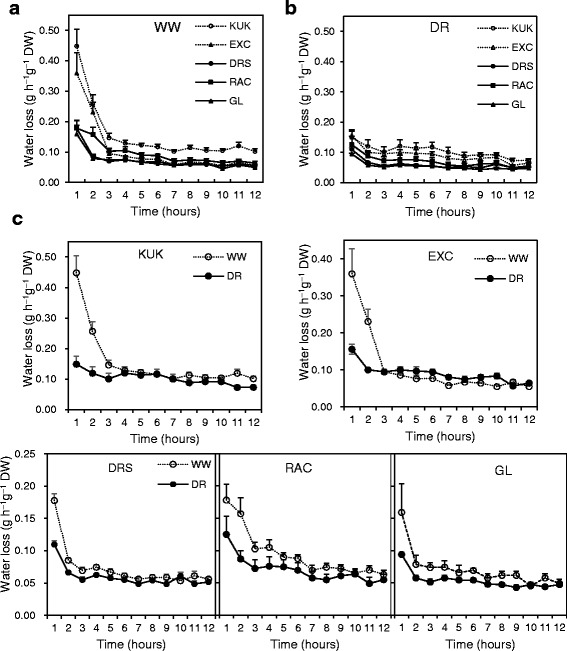
Water loss rates of flag leaves detached from five wheat lines grown under well-watered (WW) conditions and drought (DR). Water loss rates of flag leaves detached from (**a**) WW and (**b**) DR plants over 12 h. **c** Comparison of water loss rates of WW and DR plants. KUK - Kukri, EXC - Excalibur, DRS - Drysdale, RAC - RAC875, GL - Gladius. Water loss rates were expressed as weight loss per hour per dry weight (DW) of flag leaves. Means and standard errors (indicated by bars) were calculated from four replicates

In order to determine the cuticle’s ability to act as a barrier against water loss, water losses were assessed using detached flag leaves of the five wheat lines, which were grown under well-watered (WW) conditions or under drought (DR). As shown in Fig. 1a, during the first 2 hours of dehydration the WW Kukri and Excalibur leaves lost water more rapidly than leaves of RAC875, Drysdale and Gladius. After 2 hours, the water loss rates became stable in all five lines and could be considered residual transpiration rates (RTRs), which are primarily contributed by the cuticle. The RTRs of WW Kukri plants were higher than those of the other four lines (Fig. 1a) (*P* = <0.001, Additional file 2). Water loss rates from flag leaves detached from plants grown under DR were relatively stable during the entire 12 h-long test (Fig. 1b). Similar to results obtained for WW wheat plants, the RTRs of Kukri plants grown under drought conditions were higher than those of DR RAC875, Drysdale or Gladius (Fig. 1b), however the difference was less pronounced compared to the WW experiments (Fig. 1a).

Comparison of RTRs of each line under WW compared with DR conditions revealed that RTRs of Kukri, Drysdale, RAC875 and Gladius were lower (*P* = <0.001, Additional file 2) under drought whereas the RTR of Excalibur was higher under drought compared with WW conditions (*P* = 0.001, Additional file 2) (Fig. 1c).

### Investigation of stomatal density

**Fig. 2:**
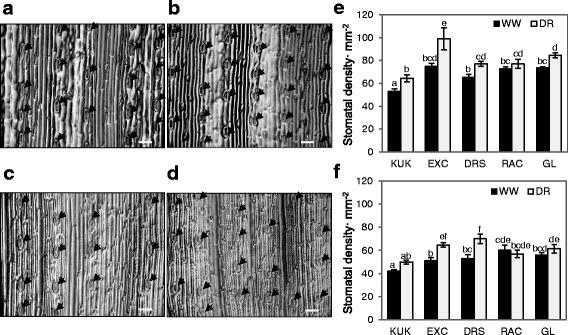
Images of stomata on the epidermis of flag leaves of investigated wheat lines. **a** Stomata on the adaxial side of a flag leaf in Kukri. Stomata are indicated by arrows. **b** Stomata on the adaxial side of a flag leaf in Gladius. **c** Stomata on the abaxial side of a flag leaf in Kukri. **d** Stomata on the abaxial side of a flag leaf in Gladius. **e** Calculated stomatal density on the adaxial side of flag leaves. **f** Calculated stomatal density on the abaxial side of flag leaves. KUK - Kukri, EXC - Excalibur, DRS - Drysdale, RAC - RAC875, GL - Gladius, WW - well-watered conditions, DR - drought. Means and standard errors were calculated from five plants. Different letters on top of error bars indicate significant difference at *P* < 0.05. Scale bars represent 50 μm

Stomata are specialized structures on leaf surfaces that control the exchange of water and gases between plants and the environment and therefore stomatal density may affect the rate of leaf water loss. More stomata per unit of leaf area existed on the adaxial sides of flag leaves than on the abaxial sides, as shown in the representative non-glaucous and drought sensitive cultivar Kukri (Fig. 2a, c) and the glaucous and drought tolerant Gladius (Fig. 2b, d). Kukri had the lowest number of stomata per unit of leaf surface area on both surfaces of leaves under both control and drought treatment (Fig. 2e, f). RAC875 was the only line in which no significant differences in stomatal density were observed between treatments on both leaf sides. Overall, the stomatal density on both sides of flag leaves collected from plants grown under well-watered conditions in the other four lines (Kukri, Excalibur, Drysdale and Gladius) was 9.6–32.2% lower than that of leaves of plants subjected to drought.

### Microscopic assessments of leaf surfaces

**Fig. 3:**
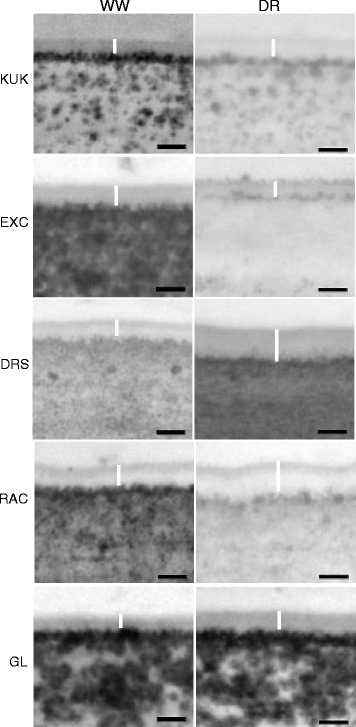
Transmission electron micrographs of cuticle layers on the abaxial epidermis of flag leaves in five wheat lines. KUK - Kukri, EXC - Excalibur, DRS - Drysdale, RAC - RAC875, GL - Gladius, WW - well-watered conditions, DR - drought. Cuticle layers are marked using *white* lines. Scale bars = 100 nm

The thickness of the cuticle layer was examined on the abaxial side of flag leaves grown under the same conditions as before using transmission electron microscopy. Under WW conditions, modest differences were found in the thickness of the cuticle layer amongst the five genotypes, whereas differences between WW and DR within lines were larger and more obvious (Fig. 3). In particular, the thickness was greatly increased by drought on the abaxial side of Drysdale and RAC875 leaves, slightly increased in Kukri and Gladius, and decreased in Excalibur.

**Fig. 4:**
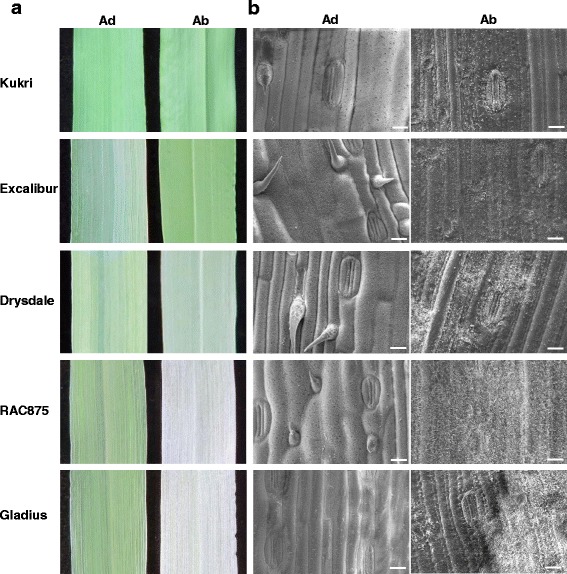
Visualisation of epicuticular waxes on the flag leaves of five wheat lines. **a** Wax phenotypes on the adaxial (Ad) and abaxial (Ab) sides of flag leaves of Kukri, Excalibur, Drysdale, RAC875 and Gladius. **b** Scanning electron micrographs of the epicuticular waxes on the adaxial (Ad) and abaxial (Ab) sides of flag leaves of Kukri, Excalibur, Drysdale, RAC875 and Gladius. Scale bars = 20 μm

**Fig. 5:**
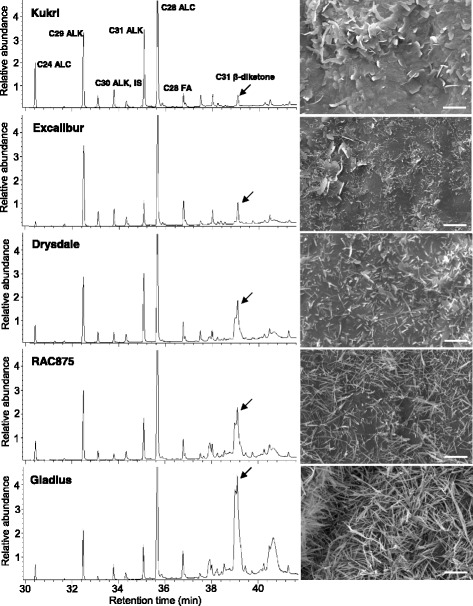
GC-MS chromatograms representing biochemical compositon of extracted cuticular waxes and SEM images of wax crystalloids on the abaxial side of the flag leaf in the five wheat lines examined. ALC - primary alcohol, ALK – alkane, FA - fatty acid, IS – internal standard. Scale bars = 5 μm. Arrows indicate C31 β-diketone

Accumulation of epicuticular waxes on leaf surfaces confers a whitish and powdery appearance to most modern wheat varieties termed glaucousness. Kukri is a non-glaucous, glossy cultivar, Excalibur has a very low level and Drysdale a medium level of glaucousness, while RAC875 and Gladius were the most glaucous lines examined (Fig. 4a). Variations in glaucousness were more noticeable on the abaxial sides of leaves than on the adaxial sides. When examined under SEM, it was found that wax distribution and structure were different on the adaxial and abaxial sides of leaves. Compared with variable waxes on the abaxial side, the adaxial side waxes were more densely and evenly deposited (Fig. 4b). The specific structures of waxes were explored under high magnification. No difference was found on the adaxial sides of leaves: the dense platelet- shaped wax crystalloids were dominant in all five lines (Additional file 3). However, different wax shapes were observed on the abaxial side of leaves. The plate-shaped wax crystalloids prevailed on the abaxial side of flag leaves of Kukri and Excalibur, a mixture of plates and tubular shapes were observed in Drysdale, while long tubule-like crystalloids were the dominant wax structures on the surfaces of the RAC875 and Gladius leaves (Fig. 5). Platelets, supposedly composed of primary alcohols and alkanes, and tubules, attributed to β-diketones [14], are the most frequent types of wax crystalloids found in plants according to the classification and terminology proposed by Barthlott et al. [41].

### Genotypic variations in epicuticular wax composition and responses to water deficit

To investigate the association between water loss rates and epicuticular waxes, we analysed the amount and composition of epicuticular waxes on the flag leaves of these five wheat lines using gas chromatography in combination with mass spectrometry (GC-MS). We observed a good correlation in these lines between the content of β-diketones and the predominant presence of long tubule-like crystalloids in wax layers (Fig. 5). The specific amounts of cuticular waxes of Kukri and RAC875 were previously presented [16]. Data shown here for Excalibur, Drysdale and Gladius were generated from wheat plants grown under exactly the same conditions as Kukri and RAC875, described by Bi et al. [16].

**Fig. 6:**
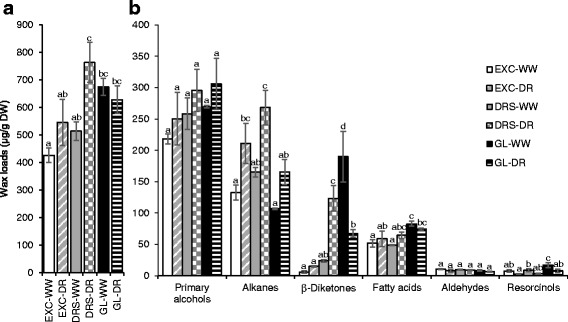
Wax amounts and composition on flag leaves of wheat lines grown under well-watered (WW) conditions and drought (DR). **a** Total wax loads. **b** Contents of each wax species. EXC - Excalibur, DRS - Drysdale, GL - Gladius. Means and standard errors (indicated by bars) were calculated from three replicates. Different small letters on top of error bars indicate significant differences at *P* < 0.05. Wax loads were calculated per gram of dry leaf weight (DW)

Under WW conditions, Gladius had the highest total wax loads on flag leaves; the total amounts of waxes decreased in the order of Drysdale and Excalibur (Fig. 6a). In terms of wax composition, primary alcohols were the most abundant wax species in all three wheat lines, comprising from 40.0 to 51.1% of total wax loads (Fig. 6b). The second most abundant wax species were C31 β-diketone in Gladius (28.2%) and alkanes for the other two cultivars. In Excalibur and Drysdale, the contents of β-diketones were much lower, accounting for 1.4% and 4.6% of total wax loads, respectively. The contents of alkanes in the total flag leaf waxes of Excalibur, Drysdale and Gladius were 31.1%, 32.1% and 15.9%, respectively. Among minor components of waxes, which were present on leaves of all examined lines, were fatty acids (9.5–12.3%), aldehydes (1.2–2.4%) and resorcinols (1.7–2.5%).

When comparing total wax loads of plants grown under WW and DR conditions, we found that drought significantly increased total wax loads on flag leaves of Drysdale by 48.5% (Fig. 6a). The wax load was moderately increased on leaves of Excalibur (28.0%) and slightly decreased on leaves of Gladius (−6.8%) (Fig. 6a). The contents of alkanes were increased in all three wheat lines under drought while the amounts of β-diketones were increased in Drysdale but decreased in Gladius (Fig. 6b).

**Fig. 7:**
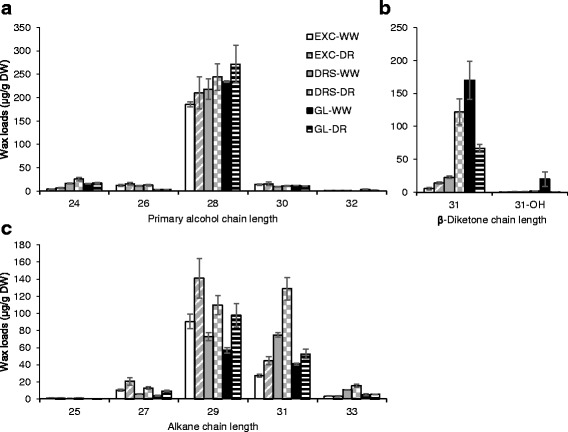
Carbon chain length distribution of major cuticular wax classes on flag leaves of wheat lines grown under well-watered (WW) conditions and drought (DR). **a** Carbon chain lengths of primary alcohols. **b** Carbon chain length of β-diketones. **c** Carbon chain lengths of alkanes. EXC - Excalibur, DRS - Drysdale, GL - Gladius. Means and standard errors (indicated by bars) were calculated from three replicates. Wax loads were calculated per gram of dry leaf weight (DW). Tiny amounts of 20-, 22- and 34-carbon primary alcohols, and 23-carbon alkane are not shown but used for calculation of total wax loads in Fig. 6

The distribution of carbon chain lengths of major wax species extracted from flag leaves of wheat lines were examined (Fig. 7). The carbon chain lengths of primary alcohols (Fig. 7a) range from 20 to 34, the β-diketone (Fig. 7b) is 31 carbon-long and alkanes (Fig. 7c) range from 23 to 33. The four most abundant wax components on flag leaves were C28 primary alcohol, C31 β-diketone, and C29 and C31 alkanes. Statistical analyses of cuticular waxes within major wax species can be found in Additional file 4 and the carbon chain length distribution of three minor wax species (fatty acids, aldehydes and resorcinols) can be found in Additional file 5.

**Fig. 8:**
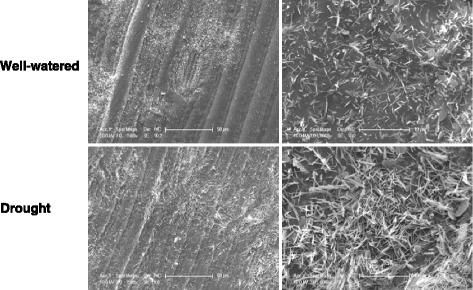
SEM images of abaxial flag leaf surfaces of cv. Drysdale grown under well-watered and drought conditions at 1500X magnification (*left column*) and 8000X magnification (*right column*)

Increases in the amounts of epicuticular waxes under drought were also observed by SEM in the most drought-responsive cultivar, Drysdale (Fig. 8). The SEM image under high magnification shows significant accumulation of the tubule-shaped crystalloids under drought, correlating with the drought-induced increase of β-diketone in this cultivar as revealed by GC-MS analysis (Fig. 6).

## Discussion

Glaucousness is an easy trait to select for in a breeding program since it can be readily scored visually. Consequently, if this trait were of major importance for adaptation to dry environments, we would expect to see almost exclusive adoption of glaucous varieties in these environments. However, this is not the case for wheat in drought-prone regions such as southern Australia where we see a full range of glaucousness, from low to high, in modern varieties. On the other hand, a number of studies have shown that cuticle modifications could significantly increase plant drought tolerance. A possible explanation for this apparent contradiction is that glaucousness is only one aspect of cuticle composition and structure and other properties or changes in the cuticle in response to stress may also be important. In this study we sought to tease apart different aspects of the leaf surface structure and cuticle composition of five Australian wheat lines with a range of leaf glaucousness and different cuticular responses under water-limited conditions. These genotypes were selected for this study because they were all bred for a similar production environment but differed in their responses to drought and related environmental factors, including heat, salt and nutrient stresses.

In previous studies it was found that the cuticle contributed more than 50% of total transpiration when wheat plants were stressed [13], and that the residual transpiration rate (RTR), defined as water loss from detached leaves at minimal stomatal aperture, plays an important role in plant survival under severe water deficit. In our water loss tests the RTRs of Kukri, the least drought tolerant and non-glaucous line, were significantly higher than those of the other more glaucous lines Gladius, RAC875 and Drysdale. The trend was observed irrespective of whether leaves used in the experiment were detached from plants cultivated under sufficient or water limited conditions (Fig. 1a, b). Interestingly, the less glaucous cultivar Excalibur (with intermediate glaucousness between the glossy Kukri and the other more glaucous lines (Figs 5 and 6)), demonstrated RTRs similar to the glaucous RAC875 under well-watered conditions and similar to the glossy line Kukri under drought (Fig. 1a and b). There was a difference between responses to the treatment within lines for RTR, such that the RTR of Excalibur leaves increased under drought, while the RTRs of all other lines decreased with treatment. This fits with the previous observations that Excalibur shows an adaptive response to drought [22]. The water loss rate of detached leaves is not an indicator of drought tolerance, defined here as the ability of a cultivar to maintain yield under field conditions where water availability limits yield to below 2 t/ha. Glaucousness, however, has previously been associated with improved grain yield in both irrigated bread wheat [42], and in barley [19] and durum wheat [24] grown in water-limited environments. In 16 durum wheat genotypes and barley isolines, glaucousness did not significantly affect RTRs or cuticular conductance [19, 24]. It has been suggested, therefore, that the association between glaucousness and yield is an indirect result of improved transpiration efficiency under conditions of water limitation and/or high evaporative demand. Here we found that RTRs among genotypes with a range of glaucousness did differ significantly with drought treatment, but that there was no straightforward correlation (either positive or negative) between visible glaucousness and the response to stress. These results suggest that factors contributing to the visible glaucous phenotype, rather than glaucousness itself, may influence drought response.

Over the past few years there have been various reports on wheat cuticle and glaucousness related genes, and their involvement in protection from biotic and abiotic stresses [14, 15, 21, 43–47]. The genetic control of wheat waxiness is complex with *W1* and *W2* loci (*W*: wax production gene) contributing to, and *Iw1* and *Iw2* inhibiting glaucousness in near-isogenic lines. When both functional alleles of W1 and W2 were present, lines had significantly lower rates of water loss and chlorophyll leaching than the non-glaucous and other glaucous NILs (*W1w2* and *w1W2*) [14]. Again, this was not the result of glaucousness per se, but was associated with the production and hydroxylation of the cuticle component wax β-diketone [14, 15]. Here, we showed a correlation between the amounts of β-diketones and long tubule-like wax crystalloids and thus the level of glaucousness in five wheat lines (Figs. 4 and 5). However, the increase in both β-diketones and total leaf wax loads observed with drought treatment in the cultivar Drysdale was not found in Excalibur or Gladius (Fig. 6). There was no significant effect of the treatment on β-diketone quantity but a significant interaction between cultivar and treatment was observed (Additional file 4). This indicated that quantitative differences in this cuticle wax component alone, whilst contributing to glaucousness, could not explain different stress responses.

In our experiments, the accumulation of free fatty acids remained mostly unaffected by drought, while alkanes increased significantly in all tested wheat lines (Fig. 6 and [16]). These data are in agreement with results from previous studies. Fatty acids create relatively poor hydrophobic barriers to water diffusion through both natural and artificial cuticular membranes. In contrast, alkanes represent an effective water permeability barrier [48, 49]. The activation by water stress of biochemical pathways providing components for the biosynthesis of alkanes has been demonstrated for a number of plant species [12, 50, 51].

Growing wheat plants under conditions of limited water did not influence the morphology of wax crystalloids on the surfaces of flag leaves, although an increase in the density of waxes was observed (Fig. 8). However, a significant increase of cuticle thickness was seen for two of the five lines, Drysdale and RAC875 (Fig. 3). In addition to the highest drought-induced increases in cuticle thickness, these two lines also demonstrated the most significant increases in total wax loads (48.5% and 31.5%, respectively) during plant growth under drought (Fig. 6 and [16]). In contrast, we observed a discrepancy between these two parameters for the cuticle of flag leaves of Excalibur (28% increase in amount of waxes but a decrease in cuticle thickness on the abaxial side of the leaf) and Gladius (6.8% decrease in wax load versus an increase in cuticle thickness on the abaxial side of the leaf). These apparent inconsistencies could reflect different responses in the abaxial versus adaxial wax deposition, or the lack of regulation of wax deposition in response to environmental stress.

Enhancement of leaf cuticular wax production and increases in cuticle thickness appear to represent a prevalent response to water deficit across the terrestrial plant kingdom [12]. For example, a 75% increase in total wax amount per unit of leaf area and 49% increase in cuticle thickness were reported in *Arabidopsis* under water deficit [49], and, on leaves of tree tobacco, drought induced over 150% increase in total wax accumulation [52]. The changes in *Arabidopsis* cuticle were associated with decreased cuticle permeability measured as reductions in water loss and chlorophyll leaching rates of detached leaves [49]. In addition, elevated cuticle membrane thickness in the *Arabidopsis cer9* (*eceriferum 9*) mutant correlated with lower transpiration rates and improved water use efficiency [53]. Synthesis of a larger cutin framework was proposed as a mechanism of drought acclimatisation for *Arabidopsis* [49], whereas the major water permeability barrier of tomato fruit cuticle was found to be intracuticular rather than epicuticular wax [26].

Both cuticle thickness and stomatal density appeared to be relevant contributors to the drought tolerance of four European winter wheat cultivars with contrasting behaviour under limited water supply, with the most drought tolerant cultivar demonstrating the lowest stomatal density on both flag leaf surfaces [54]. In our work, however, the drought-sensitive cultivar Kukri, which in leaf desiccation tests had the highest water loss rates compared to the other four wheat lines, had the lowest stomatal density on both leaf surfaces (Figs. 1 and 2). Our findings were consistent with those of Shahinnia et al. [55] who investigated stomatal traits in the parents and a genetic mapping population from a cross between RAC875 and Kukri. They found that RAC875 had a higher density of smaller stomata than Kukri, so that the total pore surface per unit leaf area did not differ between these two genotypes. Together with co-located quantitative trait loci for stomatal traits and yield in this population, their results suggested that the stomatal density-size relationship contributed to the higher yield under drought of RAC875 compared with Kukri. That is, consistent water use due to the higher density but smaller stomatal pore aperture might be beneficial in water-limited environments. In our experiments, all other lines had a higher stomatal density than Kukri but the total pore area per unit of leaf was not measured. Nonetheless, drought treatment increased stomatal density in Kukri, Excalibur, Drysdale and Gladius, but not in RAC875. Xu and Zhou [56] found that moderate water deficits increased, while more severe water deficits reduced, stomatal density on leaves of a perennial grass, *Leymus chinensis*. Their results also indicated that an increase in leaf stomatal density was positively associated with leaf–level water use efficiency. When assessed over the entire desiccation period, including both stomatal (first 2 hours) and residual transpiration (thereafter), the difference between control and drought-treated leaves was not significant for the cultivar Excalibur. In contrast, Excalibur showed the greatest difference in leaf stomatal density with treatment. Although transpiration efficiency was not directly measured in our experiments, there was no indication that differences in stomatal density under drought explained differences in water loss rates in these lines.

Overall, these results suggested that glaucousness was defined by β-diketone levels, but that its precise value for drought tolerance remains unresolved. The small but obvious changes in RTRs of wheat lines under drought, especially in RTRs of Drysdale and RAC875, corresponded well with their levels of drought-induced wax accumulation and thickening of cuticle layers. However, there was no similar relationship found between stomatal density and water loss for the examined lines, and no meaningful changes occurred in stomatal numbers during plant growth under drought.

## Conclusions

The basis for this study was to develop an understanding of cuticle structure and deposition of wheat in relation to drought tolerance since there is some evidence, and a reasonable physiological expectation, that the cuticle will play an important role in regulating water loss. Although varying levels of glaucousness are found in modern wheat varieties and glaucousness is an easy visual trait to score, few breeders would regard it as an important selection target. Overall, our results show that considerable variation exists between genotypes in the composition and structure of the cuticle. The relationship between the cuticle and drought tolerance is not simple and glaucousness is not a unique indicator of tolerance. Rather, specific wax composition, in particular the presence of significant proportions of C31 β-diketone, adaptive changes in composition and amount induced by exposure to drought and wax crystal structure will all influence leaf water loss under conditions of water deficit. These features of cuticle composition and deposition appear important and should provide useful selection criteria although they are more difficult to measure than glaucousness.

## Additional files

Additional file 1: Figure S1. Soil water tension monitored for well-watered and drought conditions. Soil water tension at 10 cm and 30 cm depths were shown. (PDF 37 kb)

Additional file 2: Table S1. Results of statistical analyses for water loss rates test in Fig. 1. (PDF 101 kb)

Additional file 3: Figure S2. Scanning electron micrographs of epicuticular waxes on the adaxial side of flag leaves in five wheat lines. (A) Kukri; (B) Excalibur; (C) Drysdale; (D) RAC875 and (E) Gladius. Scale bars represent 5 μm. (PDF 194 kb)

Additional file 4: Statistical analyses for cuticular waxes within each of major wax species in Fig. 7. (PDF 68 kb)

Additional file 5: Figure S3. Carbon chain length distribution of minor cuticular wax species on flag leaves of wheat lines grown under well-watered (WW) condition and drought (DR). (A) Carbon chain length of fatty acids. (B) Carbon chain length of aldehydes. (C) Carbon chain length of resorcinols. EXC - Excalibur, DRS - Drysdale, GL - Gladius. Means and standard errors (indicated by bar) were calculated from three replicates. Wax loads were calculated per gram of dry leaf weight (DW). (PDF 96 kb)

## Abbreviations

ALC: Primary alcohol
ALK: Alkane
DR: Drought
DRS: Drysdale
EXC: Excalibur
FA: Fatty acid
GC-MS: Gas chromatography-mass spectrometry
GL: Gladius
IS: Internal standard
KUK: Kukri
RAC: RAC875
RTR: Residual transpiration rate
SEM: Scanning electron microscopy
TEM: Transmission electron microscopy
VLCFA: Very long chain fatty acid
WW: Well-watered; DR: drought.
