# Supplementary figures and images for: The impact of drought on wheat leaf cuticle properties
Source: BMC Plant Biol. 2017 May 8;17:85. doi: 10.1186/s12870-017-1033-3 (PMC5422891; doi:10.1186/s12870-017-1033-3)

Well-watered conditions

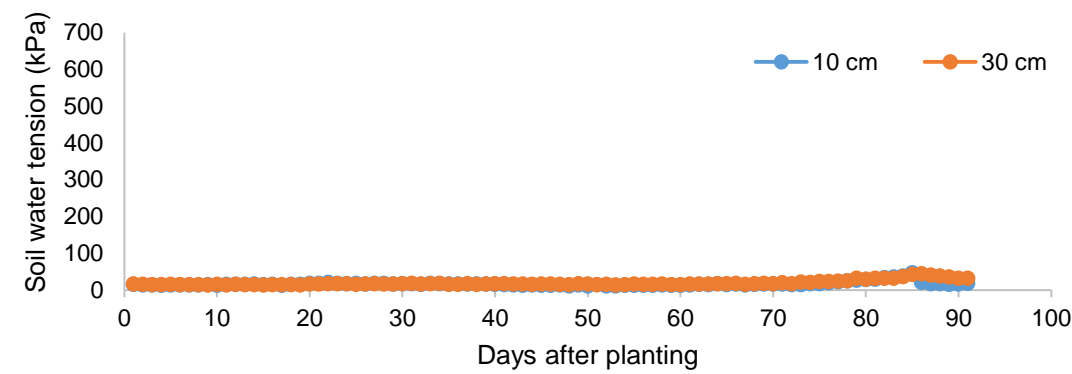

Drought

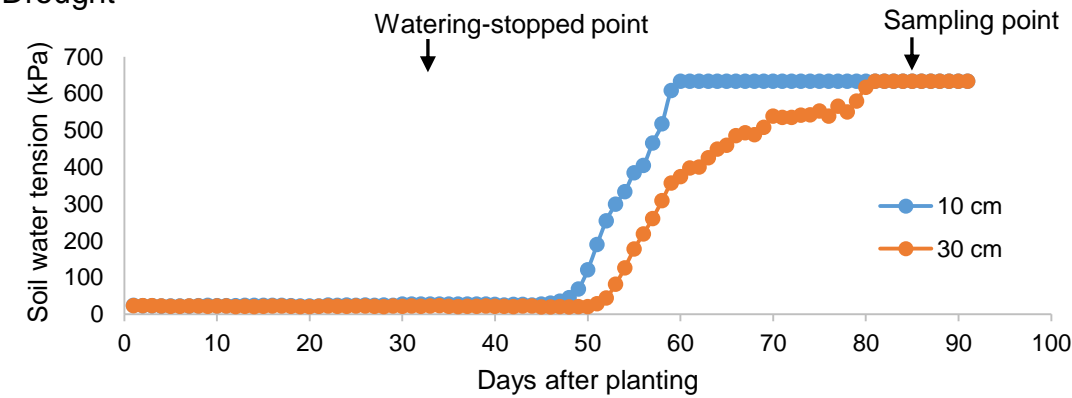

Supplement: Supplementary file 1 — Soil water tension monitored for well-watered and drought conditions. Soil water tension at 10 cm and 30 cm depths were shown. (PDF 37 kb) [file 12870_2017_1033_MOESM1_ESM.pdf]

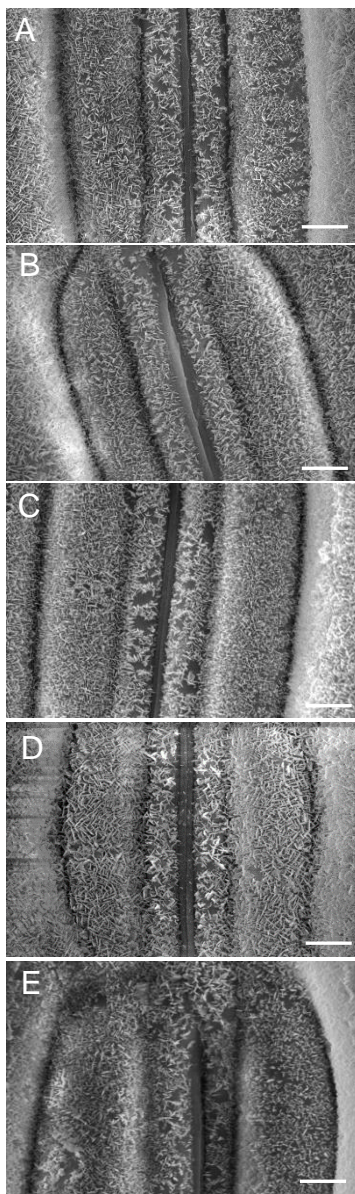

Supplement: Supplementary file 3 — Scanning electron micrographs of epicuticular waxes on the adaxial side of flag leaves in five wheat lines. (A) Kukri; (B) Excalibur; (C) Drysdale; (D) RAC875 and (E) Gladius. Scale bars represent 5 μm. (PDF 194 kb) [file 12870_2017_1033_MOESM3_ESM.pdf]

A

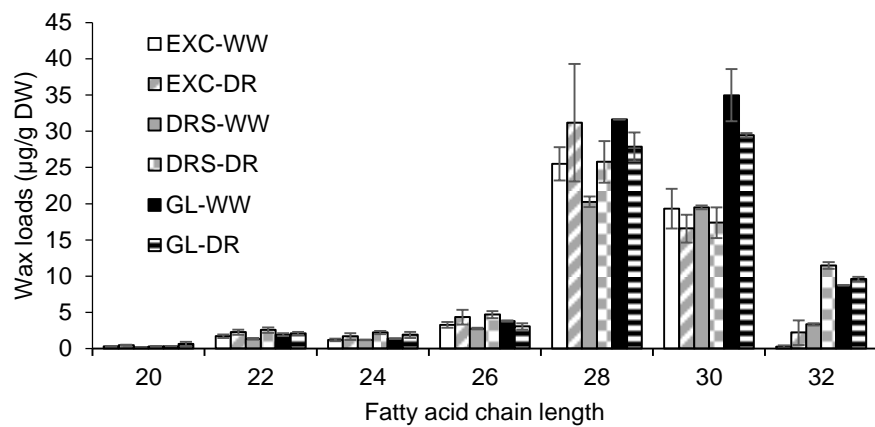

B

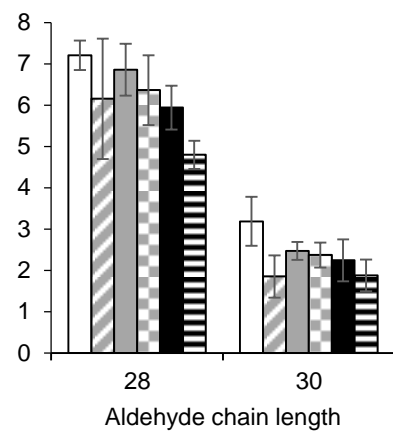

C

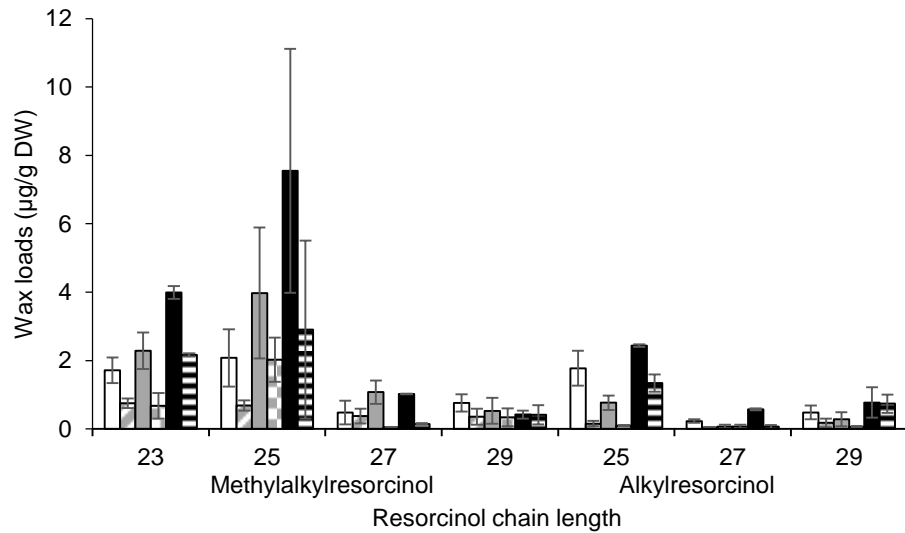

Supplement: Supplementary file 5 — Carbon chain length distribution of minor cuticular wax species on flag leaves of wheat lines grown under well-watered (WW) condition and drought (DR). (A) Carbon chain length of fatty acids. (B) Carbon chain length of aldehydes. (C) Carbon chain length of resorcinols. EXC - Excalibur, DRS - Drysdale, GL - Gladius. Means and standard errors (indicated by bar) were calculated from three replicates. Wax loads were calculated per gram of dry leaf weight (DW). (PDF 96 kb) [file 12870_2017_1033_MOESM5_ESM.pdf]
