# Supplementary material for: The impact of drought on wheat leaf cuticle properties
Source: BMC Plant Biol. 2017 May 8;17:85. doi: 10.1186/s12870-017-1033-3 (PMC5422891; doi:10.1186/s12870-017-1033-3)
Supplement: Supplementary file 2 — Results of statistical analyses for water loss rates test in Fig. 1. (PDF 101 kb) [file 12870_2017_1033_MOESM2_ESM.pdf]

Summary table of General Linear Model repeated measures ANOVA (RTR from 3h) for Fig 1A and B.

| Source | Type II sums of Square | df | Mean Square | F | Sig. |
|---|---|---|---|---|---|
| Intercept | 2.168 | 1 | 2.168 | 1346.856 | .000 |
| Treatment | 0.007 | 1 | 0.007 | 4.502 | .042 |
| Genotype | 0.134 | 4 | 0.033 | 20.752 | .000 |
| Treatment*Genotype | 0.013 | 4 | 0.003 | 1.968 | .125 |
| Error | 0.048 | 30 | 0.002 | | |

ANOVA single factor – treatment (RTR from 3h) for Fig 1C.

| Genotype | P value |
|---|---|
| Gladius | 0.000 |
| Kukri | 0.001 |
| RAC875 | 0.000 |
| Excalibur | 0.001 |
| Drysdale | 0.001 |
