# Supplementary material for: The impact of drought on wheat leaf cuticle properties
Source: BMC Plant Biol. 2017 May 8;17:85. doi: 10.1186/s12870-017-1033-3 (PMC5422891; doi:10.1186/s12870-017-1033-3)
Supplement: Supplementary file 4 — Statistical analyses for cuticular waxes within each of major wax species in Fig. 7. (PDF 68 kb) [file 12870_2017_1033_MOESM4_ESM.pdf]

```
GLM FAC26 FAC28 FAC30 FAC32 BY Treatment Plantname
 /METHOD=SSTYPE(3)
 /INTERCEPT=INCLUDE
 /POSTHOC=Treatment Plantname(LSD)
 /CRITERIA=ALPHA(.05)
 /DESIGN= Treatment Plantname Treatment*Plantname.
```

General Linear Model

Notes

Output Created		28-FEB-2017 11:26:06
Comments		
Input	Data	/Volumes/home/penny.tricker/My Documents/Supervision/Huihui Bi/Cuticle ms/Revision March 2017/Figure7 data.sav
	Active Dataset	DataSet1
	Filter	<none>
	Weight	<none>
	Split File	<none>
	N of Rows in Working Data File	18
Missing Value Handling	Definition of Missing	User-defined missing values are treated as missing.
	Cases Used	Statistics are based on all cases with valid data for all variables in the model.
Syntax		GLM FAC26 FAC28 FAC30 FAC32 BY Treatment Plantname /METHOD=SSTYPE(3) /INTERCEPT=INCLUDE /POSTHOC=Treatment Plantname(LSD) /CRITERIA=ALPHA(.05) /DESIGN= Treatment Plantname Treatment*Plantname.
Resources	Processor Time	00:00:00.02
	Elapsed Time	00:00:00.00

Warnings

Post hoc tests are not performed for Treatment because there are fewer than three groups.

Between-Subjects Factors

		N
Treatment	dr	9
	ww	9
Plant name	Drysdale	6
	Excalibur	6
	Gladius	6

Multivariate Tests[a]

Effect		Value	F	Hypothesis df	Error df
Intercept	Pillai's Trace	.970	72.719 [b]	4.000	9.000
	Wilks' Lambda	.030	72.719 [b]	4.000	9.000
	Hotelling's Trace	32.320	72.719 [b]	4.000	9.000
	Roy's Largest Root	32.320	72.719 [b]	4.000	9.000
Treatment	Pillai's Trace	.768	7.457 [b]	4.000	9.000
	Wilks' Lambda	.232	7.457 [b]	4.000	9.000
	Hotelling's Trace	3.314	7.457 [b]	4.000	9.000
	Roy's Largest Root	3.314	7.457 [b]	4.000	9.000
Plantname	Pillai's Trace	1.581	9.428	8.000	20.000
	Wilks' Lambda	.040	9.036 [b]	8.000	18.000
	Hotelling's Trace	8.547	8.547	8.000	16.000
	Roy's Largest Root	5.900	14.751 [c]	4.000	10.000
Treatment * Plantname	Pillai's Trace	.994	2.470	8.000	20.000
	Wilks' Lambda	.174	3.148 [b]	8.000	18.000
	Hotelling's Trace	3.791	3.791	8.000	16.000
	Roy's Largest Root	3.516	8.790 [c]	4.000	10.000

Multivariate Tests[a]

Effect		Sig.
Intercept	Pillai's Trace	.000
	Wilks' Lambda	.000
	Hotelling's Trace	.000
	Roy's Largest Root	.000
Treatment	Pillai's Trace	.006
	Wilks' Lambda	.006
	Hotelling's Trace	.006
	Roy's Largest Root	.006
Plantname	Pillai's Trace	.000
	Wilks' Lambda	.000
	Hotelling's Trace	.000
	Roy's Largest Root	.000
Treatment * Plantname	Pillai's Trace	.048
	Wilks' Lambda	.021
	Hotelling's Trace	.011
	Roy's Largest Root	.003

a. Design: Intercept + Treatment + Plantname + Treatment * Plantname

b. Exact statistic

c. The statistic is an upper bound on F that yields a lower bound on the significance level.

Tests of Between-Subjects Effects

Source	Dependent Variable	Type III Sum of Squares	df	Mean Square
Corrected Model	FA, C26	11.707[a]	5	2.341
	FA, C28	698.383[b]	5	139.677
	FA, C30	1469.921[c]	5	293.984
	FA, C32	276.371[d]	5	55.274
Intercept	FA, C26	249.732	1	249.732
	FA, C28	13816.573	1	13816.573
	FA, C30	9721.511	1	9721.511
	FA, C32	546.352	1	546.352
Treatment	FA, C26	.872	1	.872
	FA, C28	10.473	1	10.473
	FA, C30	281.337	1	281.337
	FA, C32	30.395	1	30.395
Plantname	FA, C26	.090	2	.045
	FA, C28	233.926	2	116.963
	FA, C30	919.577	2	459.788
	FA, C32	164.621	2	82.310
Treatment * Plantname	FA, C26	10.746	2	5.373
	FA, C28	453.983	2	226.992
	FA, C30	269.007	2	134.504
	FA, C32	81.355	2	40.677
Error	FA, C26	12.388	12	1.032
	FA, C28	961.564	12	80.130
	FA, C30	666.277	12	55.523
	FA, C32	67.429	12	5.619
Total	FA, C26	273.826	18	
	FA, C28	15476.520	18	
	FA, C30	11857.710	18	
	FA, C32	890.151	18	
Corrected Total	FA, C26	24.095	17	
	FA, C28	1659.946	17	
	FA, C30	2136.199	17	
	FA, C32	343.800	17	

Tests of Between-Subjects Effects

Source	Dependent Variable	F	Sig.
Corrected Model	FA, C26	2.268	.114
	FA, C28	1.743	.199
	FA, C30	5.295	.008
	FA, C32	9.837	.001
Intercept	FA, C26	241.918	.000
	FA, C28	172.426	.000
	FA, C30	175.089	.000
	FA, C32	97.231	.000
Treatment	FA, C26	.844	.376
	FA, C28	.131	.724
	FA, C30	5.067	.044
	FA, C32	5.409	.038
Plantname	FA, C26	.043	.958
	FA, C28	1.460	.271
	FA, C30	8.281	.005
	FA, C32	14.648	.001
Treatment * Plantname	FA, C26	5.205	.024
	FA, C28	2.833	.098
	FA, C30	2.422	.131
	FA, C32	7.239	.009
Error	FA, C26		
	FA, C28		
	FA, C30		
	FA, C32		
Total	FA, C26		
	FA, C28		
	FA, C30		
	FA, C32		
Corrected Total	FA, C26		
	FA, C28		
	FA, C30		
	FA, C32		

a. R Squared = .486 (Adjusted R Squared = .272)

b. R Squared = .421 (Adjusted R Squared = .179)

c. R Squared = .688 (Adjusted R Squared = .558)

d. R Squared = .804 (Adjusted R Squared = .722)

Post Hoc Tests

Plant name

Multiple Comparisons

LSD

Dependent Variable	(I) Plant name	(J) Plant name	Mean Difference (I-J)	Std. Error	Sig.
FA, C26	Drysdale	Excalibur	-.07553569	.586599989	.900
		Gladius	.096759109	.586599989	.872
	Excalibur	Drysdale	.075535691	.586599989	.900
		Gladius	.172294801	.586599989	.774
	Gladius	Drysdale	-.09675911	.586599989	.872
		Excalibur	-.17229480	.586599989	.774
FA, C28	Drysdale	Excalibur	-5.3406818	5.16818185	.322
		Gladius	-8.7604383	5.16818185	.116
	Excalibur	Drysdale	5.34068177	5.16818185	.322
		Gladius	-3.4197565	5.16818185	.521
	Gladius	Drysdale	8.76043825	5.16818185	.116
		Excalibur	3.41975648	5.16818185	.521
FA, C30	Drysdale	Excalibur	.498731361	4.30205846	.910
		Gladius	-14.90674*	4.30205846	.005
	Excalibur	Drysdale	-.49873136	4.30205846	.910
		Gladius	-15.40548*	4.30205846	.004
	Gladius	Drysdale	14.906745*	4.30205846	.005
		Excalibur	15.405476*	4.30205846	.004
FA, C32	Drysdale	Excalibur	6.1667453*	1.36858658	.001
		Gladius	-.47101688	1.36858658	.737
	Excalibur	Drysdale	-6.166745*	1.36858658	.001
		Gladius	-6.637762*	1.36858658	.000
	Gladius	Drysdale	.471016878	1.36858658	.737
		Excalibur	6.6377622*	1.36858658	.000

Multiple Comparisons

LSD

Dependent Variable	(I) Plant name	(J) Plant name	95% Confidence Interval Lower Bound	95% Confidence Interval Upper Bound
FA, C26	Drysdale	Excalibur	-1.3536273	1.20255589
		Gladius	-1.1813325	1.37485069
	Excalibur	Drysdale	-1.2025559	1.35362727
		Gladius	-1.1057968	1.45038638
	Gladius	Drysdale	-1.3748507	1.18133247
		Excalibur	-1.4503864	1.10579678
FA, C28	Drysdale	Excalibur	-16.601183	5.91981915
		Gladius	-20.020939	2.50006267
	Excalibur	Drysdale	-5.9198192	16.6011827
		Gladius	-14.680257	7.84074445
	Gladius	Drysdale	-2.5000627	20.0209392
		Excalibur	-7.8407444	14.6802574
FA, C30	Drysdale	Excalibur	-8.8746488	9.87211152
		Gladius	-24.280125	-5.5333647
	Excalibur	Drysdale	-9.8721115	8.87464880
		Gladius	-24.778856	-6.0320961
	Gladius	Drysdale	5.53336471	24.2801250
		Excalibur	6.03209607	24.7788564
FA, C32	Drysdale	Excalibur	3.18485131	9.14863930
		Gladius	-3.4529109	2.51087712
	Excalibur	Drysdale	-9.1486393	-3.1848513
		Gladius	-9.6196562	-3.6558682
	Gladius	Drysdale	-2.5108771	3.45291087
		Excalibur	3.65586819	9.61965618

Based on observed means.
The error term is Mean Square(Error) = 5.619.

*. The mean difference is significant at the .05 level.

```
GLM AlkaneC27 AlkaneC29 AlkaneC31 AlkaneC33 BY Treatment Plantname
  /METHOD=SSTYPE(3)
  /INTERCEPT=INCLUDE
  /POSTHOC=Treatment Plantname(LSD)
  /CRITERIA=ALPHA(.05)
  /DESIGN= Treatment Plantname Treatment*Plantname.
```

General Linear Model

Notes

Output Created		28-FEB-2017 11:28:13
Comments		
Input	Data	/Volumes/home/penny.tricker/My Documents/Supervision/Huihui Bi/Cuticle ms/Revision March 2017/Figure7 data.sav
	Active Dataset	DataSet1
	Filter	<none>
	Weight	<none>
	Split File	<none>
	N of Rows in Working Data File	18
Missing Value Handling	Definition of Missing	User-defined missing values are treated as missing.
	Cases Used	Statistics are based on all cases with valid data for all variables in the model.
Syntax		GLM AlkaneC27 AlkaneC29 AlkaneC31 AlkaneC33 BY Treatment Plantname /METHOD=SSTYPE(3) /INTERCEPT=INCLUDE /POSTHOC=Treatment Plantname(LSD) /CRITERIA=ALPHA(.05) /DESIGN= Treatment Plantname Treatment*Plantname.
Resources	Processor Time	00:00:00.03
	Elapsed Time	00:00:00.00

Warnings

Post hoc tests are not performed for Treatment because there are fewer than three groups.

Between-Subjects Factors

		N
Treatment	dr	9
	ww	9
Plant name	Drysdale	6
	Excalibur	6
	Gladius	6

Multivariate Tests[a]

Effect		Value	F	Hypothesis df	Error df
Intercept	Pillai's Trace	.965	61.147 [b]	4.000	9.000
	Wilks' Lambda	.035	61.147 [b]	4.000	9.000
	Hotelling's Trace	27.176	61.147 [b]	4.000	9.000
	Roy's Largest Root	27.176	61.147 [b]	4.000	9.000
Treatment	Pillai's Trace	.924	27.460 [b]	4.000	9.000
	Wilks' Lambda	.076	27.460 [b]	4.000	9.000
	Hotelling's Trace	12.204	27.460 [b]	4.000	9.000
	Roy's Largest Root	12.204	27.460 [b]	4.000	9.000
Plantname	Pillai's Trace	1.693	13.808	8.000	20.000
	Wilks' Lambda	.009	21.851 [b]	8.000	18.000
	Hotelling's Trace	33.179	33.179	8.000	16.000
	Roy's Largest Root	30.541	76.354 [c]	4.000	10.000
Treatment * Plantname	Pillai's Trace	1.205	3.790	8.000	20.000
	Wilks' Lambda	.088	5.351 [b]	8.000	18.000
	Hotelling's Trace	7.072	7.072	8.000	16.000
	Roy's Largest Root	6.564	16.409 [c]	4.000	10.000

Multivariate Tests[a]

Effect		Sig.
Intercept	Pillai's Trace	.000
	Wilks' Lambda	.000
	Hotelling's Trace	.000
	Roy's Largest Root	.000
Treatment	Pillai's Trace	.000
	Wilks' Lambda	.000
	Hotelling's Trace	.000
	Roy's Largest Root	.000
Plantname	Pillai's Trace	.000
	Wilks' Lambda	.000
	Hotelling's Trace	.000
	Roy's Largest Root	.000
Treatment * Plantname	Pillai's Trace	.007
	Wilks' Lambda	.002
	Hotelling's Trace	.000
	Roy's Largest Root	.000

a. Design: Intercept + Treatment + Plantname + Treatment * Plantname

b. Exact statistic

c. The statistic is an upper bound on F that yields a lower bound on the significance level.

Tests of Between-Subjects Effects

Source	Dependent Variable	Type III Sum of Squares	df	Mean Square
Corrected Model	Alkane C27	506.605 [a]	5	101.321
	Alkane C29	10233.303 [b]	5	2046.661
	Alkane C31	19909.968 [c]	5	3981.994
	Alkane C33	362.579 [d]	5	72.516
Intercept	Alkane C27	1967.506	1	1967.506
	Alkane C29	162941.313	1	162941.313
	Alkane C31	67908.005	1	67908.005
	Alkane C33	936.344	1	936.344
Treatment	Alkane C27	190.348	1	190.348
	Alkane C29	4467.170	1	4467.170
	Alkane C31	1945.321	1	1945.321
	Alkane C33	3.312	1	3.312
Plantname	Alkane C27	264.528	2	132.264
	Alkane C29	4305.446	2	2152.723
	Alkane C31	14983.971	2	7491.985
	Alkane C33	322.000	2	161.000
Treatment * Plantname	Alkane C27	51.729	2	25.865
	Alkane C29	1460.688	2	730.344
	Alkane C31	2980.676	2	1490.338
	Alkane C33	37.268	2	18.634
Error	Alkane C27	177.597	12	14.800
	Alkane C29	8324.655	12	693.721
	Alkane C31	2536.929	12	211.411
	Alkane C33	43.283	12	3.607
Total	Alkane C27	2651.708	18	
	Alkane C29	181499.271	18	
	Alkane C31	90354.901	18	
	Alkane C33	1342.206	18	
Corrected Total	Alkane C27	684.202	17	
	Alkane C29	18557.958	17	
	Alkane C31	22446.896	17	
	Alkane C33	405.862	17	

Tests of Between-Subjects Effects

Source	Dependent Variable	F	Sig.
Corrected Model	Alkane C27	6.846	.003
	Alkane C29	2.950	.058
	Alkane C31	18.835	.000
	Alkane C33	20.105	.000
Intercept	Alkane C27	132.942	.000
	Alkane C29	234.880	.000
	Alkane C31	321.214	.000
	Alkane C33	259.597	.000
Treatment	Alkane C27	12.862	.004
	Alkane C29	6.439	.026
	Alkane C31	9.202	.010
	Alkane C33	.918	.357
Plantname	Alkane C27	8.937	.004
	Alkane C29	3.103	.082
	Alkane C31	35.438	.000
	Alkane C33	44.636	.000
Treatment * Plantname	Alkane C27	1.748	.216
	Alkane C29	1.053	.379
	Alkane C31	7.049	.009
	Alkane C33	5.166	.024
Error	Alkane C27		
	Alkane C29		
	Alkane C31		
	Alkane C33		
Total	Alkane C27		
	Alkane C29		
	Alkane C31		
	Alkane C33		
Corrected Total	Alkane C27		
	Alkane C29		
	Alkane C31		
	Alkane C33		

a. R Squared = .740 (Adjusted R Squared = .632)

b. R Squared = .551 (Adjusted R Squared = .365)

c. R Squared = .887 (Adjusted R Squared = .840)

d. R Squared = .893 (Adjusted R Squared = .849)

Post Hoc Tests

Plant name

Multiple Comparisons

LSD

Dependent Variable	(I) Plant name	(J) Plant name	Mean Difference (I-J)	Std. Error	Sig.
Alkane C27	Drysdale	Excalibur	-6.244381*	2.22109355	.016
		Gladius	2.95133448	2.22109355	.209
	Excalibur	Drysdale	6.2443812*	2.22109355	.016
		Gladius	9.1957157*	2.22109355	.001
	Gladius	Drysdale	-2.9513345	2.22109355	.209
		Excalibur	-9.195716*	2.22109355	.001
Alkane C29	Drysdale	Excalibur	-24.360080	15.2065912	.135
		Gladius	12.9456689	15.2065912	.411
	Excalibur	Drysdale	24.3600804	15.2065912	.135
		Gladius	37.305749*	15.2065912	.030
	Gladius	Drysdale	-12.945669	15.2065912	.411
		Excalibur	-37.30575*	15.2065912	.030
Alkane C31	Drysdale	Excalibur	65.899850*	8.39465546	.000
		Gladius	55.061007*	8.39465546	.000
	Excalibur	Drysdale	-65.89985*	8.39465546	.000
		Gladius	-10.838843	8.39465546	.221
	Gladius	Drysdale	-55.06101*	8.39465546	.000
		Excalibur	10.8388432	8.39465546	.221
Alkane C33	Drysdale	Excalibur	9.6896938*	1.09649629	.000
		Gladius	8.0201091*	1.09649629	.000
	Excalibur	Drysdale	-9.689694*	1.09649629	.000
		Gladius	-1.6695847	1.09649629	.154
	Gladius	Drysdale	-8.020109*	1.09649629	.000
		Excalibur	1.66958472	1.09649629	.154

Multiple Comparisons

LSD

Dependent Variable	(I) Plant name	(J) Plant name	95% Confidence Interval Lower Bound	95% Confidence Interval Upper Bound
Alkane C27	Drysdale	Excalibur	-11.083728	-1.4050341
		Gladius	-1.8880126	7.79068160
	Excalibur	Drysdale	1.40503412	11.0837284
		Gladius	4.35636860	14.0350628
	Gladius	Drysdale	-7.7906816	1.88801265
		Excalibur	-14.035063	-4.3563686
Alkane C29	Drysdale	Excalibur	-57.492396	8.77223554
		Gladius	-20.186647	46.0779848
	Excalibur	Drysdale	-8.7722355	57.4923963
		Gladius	4.17343332	70.4380651
	Gladius	Drysdale	-46.077985	20.1866471
		Excalibur	-70.438065	-4.1734333
Alkane C31	Drysdale	Excalibur	47.6094674	84.1902335
		Gladius	36.7706243	73.3513903
	Excalibur	Drysdale	-84.190233	-47.609467
		Gladius	-29.129226	7.45153982
	Gladius	Drysdale	-73.351390	-36.770624
		Excalibur	-7.4515398	29.1292262
Alkane C33	Drysdale	Excalibur	7.30063365	12.0787540
		Gladius	5.63104894	10.4091693
	Excalibur	Drysdale	-12.078754	-7.3006337
		Gladius	-4.0586449	.719475466
	Gladius	Drysdale	-10.409169	-5.6310489
		Excalibur	-.71947547	4.05864490

Based on observed means.
The error term is Mean Square(Error) = 3.607.

*. The mean difference is significant at the .05 level.

```
GLM DiketoneC31Dik DiketoneC31OHDik BY Treatment Plantname
  /METHOD=SSTYPE(3)
  /INTERCEPT=INCLUDE
  /POSTHOC=Treatment Plantname(LSD)
  /CRITERIA=ALPHA(.05)
  /DESIGN= Treatment Plantname Treatment*Plantname.
```

General Linear Model

Notes

Output Created		28-FEB-2017 11:29:50
Comments		
Input	Data	/Volumes/home/penny.tricker/My Documents/Supervision/Huihui Bi/Cuticle ms/Revision March 2017/Figure7 data.sav
	Active Dataset	DataSet1
	Filter	<none>
	Weight	<none>
	Split File	<none>
	N of Rows in Working Data File	18
Missing Value Handling	Definition of Missing	User-defined missing values are treated as missing.
	Cases Used	Statistics are based on all cases with valid data for all variables in the model.
Syntax		GLM DiketoneC31Dik DiketoneC31OHDik BY Treatment Plantname /METHOD=SSTYPE(3) /INTERCEPT=INCLUDE /POSTHOC=Treatment Plantname(LSD) /CRITERIA=ALPHA(.05) /DESIGN= Treatment Plantname Treatment*Plantname.
Resources	Processor Time	00:00:00.01
	Elapsed Time	00:00:00.00

Warnings

Post hoc tests are not performed for Treatment because there are fewer than three groups.

Between-Subjects Factors

		N
Treatment	dr	9
	ww	9
Plant name	Drysdale	6
	Excalibur	6
	Gladius	6

Multivariate Tests[a]

Effect		Value	F	Hypothesis df	Error df
Intercept	Pillai's Trace	.887	43.305[b]	2.000	11.000
	Wilks' Lambda	.113	43.305[b]	2.000	11.000
	Hotelling's Trace	7.874	43.305[b]	2.000	11.000
	Roy's Largest Root	7.874	43.305[b]	2.000	11.000
Treatment	Pillai's Trace	.220	1.552[b]	2.000	11.000
	Wilks' Lambda	.780	1.552[b]	2.000	11.000
	Hotelling's Trace	.282	1.552[b]	2.000	11.000
	Roy's Largest Root	.282	1.552[b]	2.000	11.000
Plantname	Pillai's Trace	.899	4.903	4.000	24.000
	Wilks' Lambda	.166	8.007[b]	4.000	22.000
	Hotelling's Trace	4.637	11.594	4.000	20.000
	Roy's Largest Root	4.551	27.306[c]	2.000	12.000
Treatment * Plantname	Pillai's Trace	.874	4.661	4.000	24.000
	Wilks' Lambda	.175	7.655[b]	4.000	22.000
	Hotelling's Trace	4.439	11.098	4.000	20.000
	Roy's Largest Root	4.375	26.250[c]	2.000	12.000

Multivariate Tests[a]

Effect		Sig.
Intercept	Pillai's Trace	.000
	Wilks' Lambda	.000
	Hotelling's Trace	.000
	Roy's Largest Root	.000
Treatment	Pillai's Trace	.255
	Wilks' Lambda	.255
	Hotelling's Trace	.255
	Roy's Largest Root	.255
Plantname	Pillai's Trace	.005
	Wilks' Lambda	.000
	Hotelling's Trace	.000
	Roy's Largest Root	.000
Treatment * Plantname	Pillai's Trace	.006
	Wilks' Lambda	.001
	Hotelling's Trace	.000
	Roy's Largest Root	.000

a. Design: Intercept + Treatment + Plantname + Treatment * Plantname

b. Exact statistic

c. The statistic is an upper bound on F that yields a lower bound on the significance level.

Tests of Between-Subjects Effects

Source	Dependent Variable	Type III Sum of Squares	df	Mean Square
Corrected Model	Diketone, C31-Dik	62975.314 [a]	5	12595.063
	Diketone, C31-OH-Dik	519.344 [b]	5	103.869
Intercept	Diketone, C31-Dik	59758.012	1	59758.012
	Diketone, C31-OH-Dik	200.461	1	200.461
Treatment	Diketone, C31-Dik	199.708	1	199.708
	Diketone, C31-OH-Dik	86.662	1	86.662
Plantname	Diketone, C31-Dik	32586.515	2	16293.258
	Diketone, C31-OH-Dik	206.593	2	103.297
Treatment * Plantname	Diketone, C31-Dik	30189.090	2	15094.545
	Diketone, C31-OH-Dik	226.088	2	113.044
Error	Diketone, C31-Dik	9153.404	12	762.784
	Diketone, C31-OH-Dik	376.928	12	31.411
Total	Diketone, C31-Dik	131886.730	18	
	Diketone, C31-OH-Dik	1096.732	18	
Corrected Total	Diketone, C31-Dik	72128.718	17	
	Diketone, C31-OH-Dik	896.271	17	

Tests of Between-Subjects Effects

Source	Dependent Variable	F	Sig.
Corrected Model	Diketone, C31-Dik	16.512	.000
	Diketone, C31-OH-Dik	3.307	.042
Intercept	Diketone, C31-Dik	78.342	.000
	Diketone, C31-OH-Dik	6.382	.027
Treatment	Diketone, C31-Dik	.262	.618
	Diketone, C31-OH-Dik	2.759	.123
Plantname	Diketone, C31-Dik	21.360	.000
	Diketone, C31-OH-Dik	3.289	.073
Treatment * Plantname	Diketone, C31-Dik	19.789	.000
	Diketone, C31-OH-Dik	3.599	.060
Error	Diketone, C31-Dik		
	Diketone, C31-OH-Dik		
Total	Diketone, C31-Dik		
	Diketone, C31-OH-Dik		
Corrected Total	Diketone, C31-Dik		
	Diketone, C31-OH-Dik		

a. R Squared = .873 (Adjusted R Squared = .820)

b. R Squared = .579 (Adjusted R Squared = .404)

Post Hoc Tests

Plant name

Multiple Comparisons

LSD

Dependent Variable	(I) Plant name	(J) Plant name	Mean Difference (I-J)	Std. Error
Diketone, C31-Dik	Drysdale	Excalibur	53.853042*	15.9455706
		Gladius	-50.34907*	15.9455706
	Excalibur	Drysdale	-53.85304*	15.9455706
		Gladius	-104.2021*	15.9455706
	Gladius	Drysdale	50.349066*	15.9455706
		Excalibur	104.20211*	15.9455706
Diketone, C31-OH-Dik	Drysdale	Excalibur	.696426318	3.23577090
		Gladius	-6.8131109	3.23577090
	Excalibur	Drysdale	-.69642632	3.23577090
		Gladius	-7.509537*	3.23577090
	Gladius	Drysdale	6.81311093	3.23577090
		Excalibur	7.5095372*	3.23577090

Multiple Comparisons

LSD

Dependent Variable	(I) Plant name	(J) Plant name	Sig.	95% … Lower Bound
Diketone, C31-Dik	Drysdale	Excalibur	.005	19.1106281
		Gladius	.008	-85.091479
	Excalibur	Drysdale	.005	-88.595456
		Gladius	.000	-138.94452
	Gladius	Drysdale	.008	15.6066516
		Excalibur	.000	69.4596936
Diketone, C31-OH-Dik	Drysdale	Excalibur	.833	-6.3537128
		Gladius	.057	-13.863250
	Excalibur	Drysdale	.833	-7.7465655
		Gladius	.039	-14.559676
	Gladius	Drysdale	.057	-.23702823
		Excalibur	.039	.459398089

Multiple Comparisons

LSD

Dependent Variable	(I) Plant name	(J) Plant name	95% Confidence . Upper Bound
Diketone, C31-Dik	Drysdale	Excalibur	88.5954559
		Gladius	-15.606652
	Excalibur	Drysdale	-19.110628
		Gladius	-69.459694
	Gladius	Drysdale	85.0914794
		Excalibur	138.944521
Diketone, C31-OH-Dik	Drysdale	Excalibur	7.74656548
		Gladius	.237028229
	Excalibur	Drysdale	6.35371284
		Gladius	-.45939809
	Gladius	Drysdale	13.8632501
		Excalibur	14.5596764

Based on observed means.
The error term is Mean Square(Error) = 31.411.

*. The mean difference is significant at the .05 level.
